# Knowledge, attitudes and practices towards malaria diagnostics among healthcare providers and healthcare-seekers in Kondoa district, Tanzania: a multi-methodological situation analysis

**DOI:** 10.1186/s12936-022-04244-0

**Published:** 2022-07-21

**Authors:** Leah F. Bohle, Ally-Kebby Abdallah, Francesco Galli, Robert Canavan, Kate Molesworth

**Affiliations:** 1 Swiss Tropical and Public Health Institute, Basel, Switzerland; 2 University of Basel, Basel, Switzerland; 3 Health Promotion and System Strengthening Project (HPSS), Dodoma, Tanzania; 4 Veterinary Public Health Institute, University of Bern, Bern, Switzerland

**Keywords:** Malaria, Tanzania, Knowledge, Attitudes, Practices, Healthcare providers, Healthcare-seekers, Malaria diagnostics, Malaria rapid diagnostic test, RDT

## Abstract

**Background:**

Despite the large-scale rollout of malaria rapid diagnostic tests (RDTs) in Tanzania, many healthcare providers (HCPs) continue using blood film microscopy (BFM) and clinical examination to diagnose malaria, which can increase the risk of mal-diagnosis and over-prescribing of anti-malarials. Patients disregarding negative test results and self-treating exacerbate the problem. This study explored the knowledge, attitudes and practices of HCPs and healthcare-seekers regarding RDTs in comparison to BFM testing.

**Methods:**

A situational analysis was, therefore, conducted in Kondoa District, Dodoma Region, Tanzania. A multi-methodological approach was adopted including (i) a health facility inventory and screening of logbooks from May 2013 to April 2014 with 77,126 patient entries from 33 health facilities; (ii) a survey of 40 HCPs offering malaria services; and iii) a survey of 309 randomly selected household members from the facilities’ catchment area. Surveys took place in April and May 2014.

**Results:**

Health facility records revealed that out of 77,126 patient entries, 22% (n = 17,235) obtained a malaria diagnosis. Of those, 45% were made with BFM, 33% with RDT and 22% with clinical diagnosis. A higher rate of positive diagnoses was observed with BFM compared with RDT (71% vs 14%). In the HCP survey, 48% preferred using BFM for malaria testing, while 52% preferred RDT. Faced with a negative RDT result for a patient presenting with symptoms typical for malaria, 25% of HCPs stated they would confirm the result with a microscopy test, 70% would advise or perform a clinical diagnosis and 18% would prescribe anti-malarials. Interviews with household members revealed a preference for microscopy testing (58%) over RDT (23%), if presented with malaria symptoms. For participants familiar with both tests, a second opinion was desired in 45% after a negative microscopy result and in 90% after an RDT.

**Conclusions:**

Non-adherence to negative diagnostics by HCPs and patients continues to be a concern. Frequent training and supportive supervision for HCPs diagnosing and treating malaria and non-malaria febrile illnesses is essential to offer quality services that can instil confidence in HCPs and patients alike. The introduction of new diagnostic devices should be paired with context-specific behaviour change interventions targeting healthcare-seekers and healthcare providers.

**Supplementary Information:**

The online version contains supplementary material available at 10.1186/s12936-022-04244-0.

## Background

The new millennium saw a significant scaling up of malaria control interventions in sub-Saharan Africa, initiated by numerous international donors and in line with the United Nations Millennium Development Goals, the Roll Back Malaria Partnership objectives and the Global Technical Strategy 2016–2030 of the World Health Organization (WHO) [1–4]. Despite the success that these interventions have had on reducing, and in some cases eliminating, malaria infection related morbidity and mortality, in recent years progress has stalled calling for new, targeted and innovative measures to further the positive advancements made and to avoid regression [2, 5].

According to the 2020 World Malaria Report, the WHO African Region, accounted for ~ 94% of global malaria cases in 2019. The same year, six African countries (i.e. Nigeria, the Democratic Republic of the Congo, the United Republic of Tanzania [hereinafter Tanzania], Mozambique, Niger and Burkina Faso) accounted for half of all malaria deaths globally [2]. Tanzania has a population of almost 60 million [6]; approximately 93% of the population live in malaria transmissible areas and 96% of infections are due to *Plasmodium falciparum* [7]. The WHO estimated that there were over six million cases of malaria in Tanzania in 2019 and more than 20,000 malaria related deaths [2]. Since malaria is preventable and treatable, effective case management requires prompt access to diagnosis and treatment, provider compliance to malaria treatment guidelines and patient adherence to medication [8–10]. This in turn can help to reduce the spread of anti-malarial drug resistance, reduce unnecessary use of limited resources and better identify non-malaria febrile illnesses [8, 11]. The WHO guidelines for the treatment of malaria recommend testing all patients with suspected malaria prior to medical treatment [8]. Historically, diagnosis and treatment of malaria in Tanzanian public health facilities was presumptive [12], often leading to over diagnosis of malaria and an incorrect diagnosis for patients suffering with symptoms similar to malaria both resulting in over-prescription of anti-malarials [10, 13–15]. Fortunately, the rate of unconfirmed malaria diagnoses in Tanzania has gradually been declining, according to the Health Management Information System, from 36% in 2014 to 2% in 2018 [16].

As of 2020, diagnosis of malaria via microscopic examination in Tanzania’s public sector is available in ~ 20% of all public health facilities including regional and district hospitals and health centres [16]. However, there is a real concern of malaria being over-diagnosed when using microscopy as a sole diagnostic test [17]. The quality of malaria microscopy is dependent upon, e.g., the availability and competence of a skilled laboratory microscopist, quality assurance systems in place and electricity to power the microscope [18]. Given the simplicity and cost effectiveness of malaria rapid diagnostic tests (RDTs), together with their proven reliability, RDTs have become the most practical and suitable tool for malaria diagnosis in Tanzania, especially in remote rural areas, where resources are limited [18–24].

Previous studies in Tanzania have revealed reasonable to high levels of knowledge about the seriousness of malaria, its symptoms and preventive measures among the population and a high knowledge of the availability of artemisinin-based combination therapy (ACT) [25–31]. In contrast, and since the large-scale rollout of RDTs in Tanzania in 2009, very little research has been conducted to provide information about the knowledge, attitudes and practices towards malaria diagnostics among HCPs and healthcare-seekers alike [32–36]. The few existing studies and reports have documented a concerning disregard for negative diagnostic test results, especially when using RDTs, among healthcare-seekers and some HCPs who continue to prescribe anti-malarial medication despite negative test results [15, 36, 37]. Furthermore, two studies showed that trained HCPs appeared to be less compliant with RDT results compared with lower cadres of healthcare providers. Over-confidence in their awareness of clinical symptoms and previous experience were considered reasons for non-compliance with RDT results [10, 36, 38].

In 2011, The Health Promotion and System Strengthening (HPSS) project was launched in Tanzania. The project is implemented by the Swiss Tropical and Public Health Institute (Swiss TPH) and is funded by the Swiss Agency for Development and Cooperation (SDC). The project aims to support the Tanzanian Government to strengthen the health system countrywide and thereby advance towards Universal Health Care and Tanzania’s Development Vision 2025. The objective of the HPSS project is to apply a comprehensive approach to health system strengthening within the domains of health promotion, health financing, technology management and medicines management [39–41]. This study was conducted as part of the health promotion focal area. The aim was to describe the malaria diagnostic situation post RDT roll-out in Kondoa district and to explore healthcare providers and seekers knowledge, attitudes and practices regarding RDTs in comparison to microscopic malaria diagnostic testing.

## Methods

### Study setting

The study, here reported, was located in Kondoa District, in the north of Dodoma Region in central Tanzania. In the year prior to the study, the population was approximately 270,000 and the district was divided into 28 wards (a town, partial town or collection of villages), including 96 villages and 12 hamlets (a sub-divided village) [42]. Agriculture and livestock constituted the inhabitants’ predominant source of income. At the time, Dodoma was among the regions with a relatively low malaria prevalence with 2.5%, as reported in 2015 [43]. A recent newly proposed stratification and projection exercise on malaria risk in mainland Tanzania was more granular, classifying Kondoa District as at “low” and “very low” risk based on a combination of indicators [44].

On the national level, there was a strong framework for malaria intervention policies and strategies in place by 2014 [45] and included the free-of-charge distribution of insecticide-treated nets and long-lasting insecticidal nets (adopted in 2014), as well as a recommendation for indoor residual spraying, that was put in place in 2006. With regard to prevention and treatment, since 2001 intermittent preventive treatment is used to prevent malaria during pregnancy. While ACT is free for all ages in the public sector, the sales of oral artemisinin-based monotherapies are banned since 2006. Since 2009, patients of all ages are to receive malaria diagnostic tests and should do so free of charge in the public sector. The roll-out of RDT was completed in 2021, with repeated stock-outs between 2012 to 2015. With the introduction of malaria RTDs, the use of RDT was officially recommended as first line fever screening in all government-led facilities at all levels in 2014 [46]. Indeed, all suspected cases of malaria require confirmation by microscopy or RDT prior to treatment in both Mainland Tanzania and Zanzibar [43]. The diagnostic strategy of the National Malaria Control Programme (NMCP) aimed at achieving universal access to high quality malaria diagnostic testing in both private and public health facilities. According to the Malaria Operational Plan 2015, efforts were being made to introduce RDTs also in the private sector.

### Study overview

For this situational assessment, a multi-methodological approach was adopted and included (i) a health facility inventory and systematic screening of facility logbooks conducted over a 12-month period; (ii) a survey of healthcare providers who were offering malaria related services; and (iii) a survey of household members.

### Sample, sampling process and methodology

#### Health facility inventory

The sampling unit was the health facility. A list of all registered facilities including information on the ownership, type and serving catchment areas was established, based on information from the Kondoa District Council database. All 41 public (i.e. dispensaries, health centres and a district hospital) and private facilities (i.e. faith-based facilities, private laboratories) offering malaria counselling and diagnosis throughout Kondoa District were targeted for data collection.

Eight facilities had to be excluded owing to missing logbooks or entries, lack of readability or higher levels of confidentiality (e.g. military-run). Data collection was performed among the final sample of 33 facilities.

Data from all health facility logbooks were analysed over a 12-month period, i.e., between April/May 2013 to April/May 2014. A one-year period was chosen to provide a sound database and reflection of all seasonal fluctuations throughout the year. The total number of patients per month, the number of RDTs and blood films conducted per month, the test results, the diagnosis and treatment prescribed were counted and cross-checked by a second data collector. In addition, a retrospective and real-time stock count of available malaria tests was conducted in each of the facilities also dating back up to one year and, where available, the microscopes were tested for functionality. All counts were then entered into an Excel sheet and converted into a Stata database.

#### Survey of healthcare providers at facility level

All HCPs involved in counselling and testing for malaria at dispensary and health centre levels and two HCPs fulfilling the above criteria and randomly selected from the District Hospital, were asked to participate in the study and undergo an interview. A total of 40 HCPs were included in the study. The survey questionnaire was paper-based, semi-structured and translated into Swahili. The questionnaire included closed and few qualitative open-ended questions, which included socio-demographic questions and questions related to knowledge, attitudes and practices related to malaria diagnosis.

#### Survey of healthcare-seekers at household level

A two-stage cluster survey approach was used for the village sampling and household survey. Household members were eligible for inclusion in the study if they (i) were at least 16 years of age at the time of data collection; (ii) had undergone a malaria test within the previous 12 months at a health facility in Kondoa District at the time of data collection; (iii) were in a stable health condition; and (iv) had provided consent. The time period of last malaria test was set at 12 months to reduce the recall bias, while at the same time reflecting the seasonality of malaria and the logbook screening. The inclusion criteria (including the age limit) was shared with participants in the information and consent form; furthermore participants’ ages were again verified verbally by the research assistant. The criterion of “stable health condition” was made by each participant; however research assistants were also instructed to exclude any study participants if there were indications to suggest they might be unwell or unable to participate.

The villages were randomly selected from the catchment area of the selected health facilities. The survey team began at the centre of the village, which was divided into four main clusters. A bottle and coin method (equal to the bottle or pen method) was used to randomly select households [47]. The first household was visited; thereafter every forth household opposite the previous household was visited. This procedure was repeated until either a junction or the border of the village was reached. The bottle and coin technique was then reemployed and the process resumed until the required number of households per village had been sampled. Research assistants were asked to conduct up to 6 interviews per catchment area. The number of interviews was limited due to budget and time constraints.

The 309 household members who agreed to participate were asked to read an information sheet in Swahili and sign the consent form. Where a participant was unable to read or sign their name, an impartial witness accompanying the process, informed participants and then invited participants to provide fingerprint consent. As for the HCPs’ survey, the household survey questionnaire was paper-based, semi-structured including closed and open-ended questions and translated to Swahili.

### Data collection

A team of five Tanzanian data collectors (three female and two male) were selected. They were fully informed on the scope of the study, the ethics and use of information and consent forms. In addition, they were trained on interviewing and completing survey questionnaires, recording data, quality issue concerns and data entry. A pilot study was conducted in December 2013 to validate the survey questionnaire. Reponses to the qualitative questions were coded manually using in-vivo codes and provided as closed reply options in the final questionnaire. Final data collection took place between 6 January and 8 February 2014.

### Data analysis

Quantitative data analysis was performed with Stata, version 12, and descriptive analyses were conducted on all variables. For categorical variables, the results were reported in frequency tables with sample sizes and proportions. If a survey question allowed multiple answers, those were ranked by frequency. Continuous variables were summarized by using their mean, minimum and maximum.

Given the limited sizes of the different samples, simple exploratory methods were used to assess association between variables of interest. In the case of associations between two categorical variables, Pearson’s Chi-square test was used if the expected number of observations in all sub-categories was higher than five, whereas Fisher’s exact test was preferred if the number of expected observations in at least one sub-category was five or less. Correspondingly, p-values, here reported, were derived from Pearson’s Chi-square or Fisher’s test. If the association was significant, odds ratios (OR) were calculated and reported together with their 95% confidence interval (CI). A p-value for the odds ratio was calculated with either Pearson’s Chi-square or Fisher’s exact tests and was reported after the estimate.

When testing association between a continuous and a categorical variable, the unpaired t-test was used if the sample size of each sub-group was of at least 30 and if the observations were normally distributed (normality was assessed with the Skewness and Kurtosis test), whereas the Mann–Whitney rank sum test was used when any of the two assumptions were not true. Results were reported as group averages and the corresponding 95% CI and p-values. In case of direct comparison of proportions between two different samples, the two-sample test of proportion was used and the p-value of the Z-statistic was reported. Qualitative open-ended questions from both final survey questionnaires were coded in line with the semi-structured questionnaire, using in-vivo codes and followed by thematic grouping.

## Results

### Retrospective data collection in health facilities

#### Malaria diagnosis

Thirty-two of the 33 study health facilities provided their logbooks. These included 25 dispensaries, five health centres, one laboratory and one district hospital, reporting a total of 77,126 patient entries over a 12-month period (Table 1). Among them, 22% obtained a malaria diagnosis, 45% of all those diagnosed were tested via blood film microscopy (BFM), 33% with RDT and 22% through clinical diagnosis alone. Most patients obtained a diagnosis from the primary health care level, i.e., a dispensary (39%) or health centre (30%). The proportion of patients obtaining a malaria diagnosis out of the total number of patients visiting a facility was highest in the laboratories (57%), followed by health centres (31%), dispensaries (20%) and finally the district hospital (10%). At the district hospital, all malaria diagnoses were based on RDT. In contrast, RDTs were used less frequently in dispensaries (37%) and health centres (20%), and not at all in laboratories.

**Table 1:** Malaria diagnoses displayed in numbers and proportions, stratified by facility type, ownership and diagnostic procedure

	Overall (N = 33)	Dispensary (n = 25)	Health centre (n = 5)	District hospital (n = 1)	Laboratory (n = 1)	Government (n = 24)	Faith-based (n = 6)	Private (n = 2)
Total patients	77,126	33,439	16,587	21,430	5670	59,343	8737	9046
Total malaria diagnoses	17,235 (22.3)	6661 (19.9)	5135 (31.0)	2218 (10.3)	3221 (56.8)	9217 (15.5)	2842 (32.5)	5176 (57.2)
Positive cases	9904 (57.5)	3715 (55.8)	4021 (78.3)	64 (2.9)	2104 (65.3)	4781 (51.9)	1122 (39.5)	4001 (77.3)
RDT diagnoses	5709 (33.1)	2473 (37.1)	1018 (19.8)	2218 (100.0)	0 (0.0)	5044 (54.7)	665 (23.4)	0 (0.0)
Positive cases	786 (13.8)	500 (20.2)	222 (21.8)	64 (2.9)	–	709 (4.1)	77 (11.6)	–
Negative cases	4877 (85.4)	1940 (78.4)	783 (76.9)	2154 (97.1)	–	4300 (85.2)	577 (86.8)	–
Invalid tests	46 (0.8)	33 (1.3)	13 (1.3)	0 (0.0)	–	35 (0.7)	11 (1.7)	–
Non readable tests	0 (0.0)	0 (0.0)	0 (0.0)	0 (0.0)	–	0 (0.0)	0 (0.0)	–
Blood film/microscopy diagnoses	7795 (45.2)	1609 (24.2)	2965 (57.7)	0 (0.0)	3221 (100)	819 (8.9)	1800 (63.3)	5176 (100)
Positive cases	5518 (70.8)	754 (46.9)	2660 (89.7)	–	2104 (65.3)	725 (88.5)	792 (44.0)	4001 (77.3)
Negative cases	2226 (28.6)	821 (51.0)	288 (9.7)	–	1117 (34.7)	82 (10.0)	969 (53.8)	1175 (22.7)
Invalid tests	49 (0.6)	34 (2.1)	15 (0.5)	–	0 (0.0)	12 (1.5)	37 (2.1)	0 (0.0)
Non readable tests	2 (0.1)	0 (0.0)	2 (0.1)	–	0 (0.0)	0 (0.0)	2 (0.1)	0 (0.0)
Clinical diagnoses	3731 (21.7)	2579 (38.7)	1152 (22.4)	0 (0.0)	0 (0.0)	3354 (36.4)	377 (13.3)	0 (0.0)

The RDTs were used most frequently in government-owned facilities (n = 5044; 88%), followed by faith-based organizations (n = 665; 12%) and not used at all in the two privately owned facilities assessed. Blood film microscopy was the only test that was used in laboratories and represented 58% (n = 2965) of all tests in health centres and 24% (n = 1609) in dispensaries. Microscopy use was not reported from the district hospital. Microscopy was used exclusively by private facilities and at 63% by faith-based organization facilities, whereas government-owned facilities reported 9% use. Regardless of the diagnostic method used, the proportion of malaria positive cases diagnosed among those who received a diagnosis was 58% (Table 1) with a notably low proportion at the district hospital (3%).

The 24 government-owned health facilities conducted the highest number of malaria tests overall (n = 9217), 51% of which tested positive (Table 1), while the two private health facilities conducted 5176 malaria tests overall, with 77% testing positive. Faith-based organizations conducted the least number of malaria tests (n = 2842) and had the lowest proportion of positive diagnoses at 40%. Overall, a much higher positive test rate was observed with microscopy testing (71%) than with RDT (14%). This 57% difference (95% CI: 55.6–58.4) was found to be statistically significant (p < 0.001).

#### Malaria test availability

**Fig. 1:**
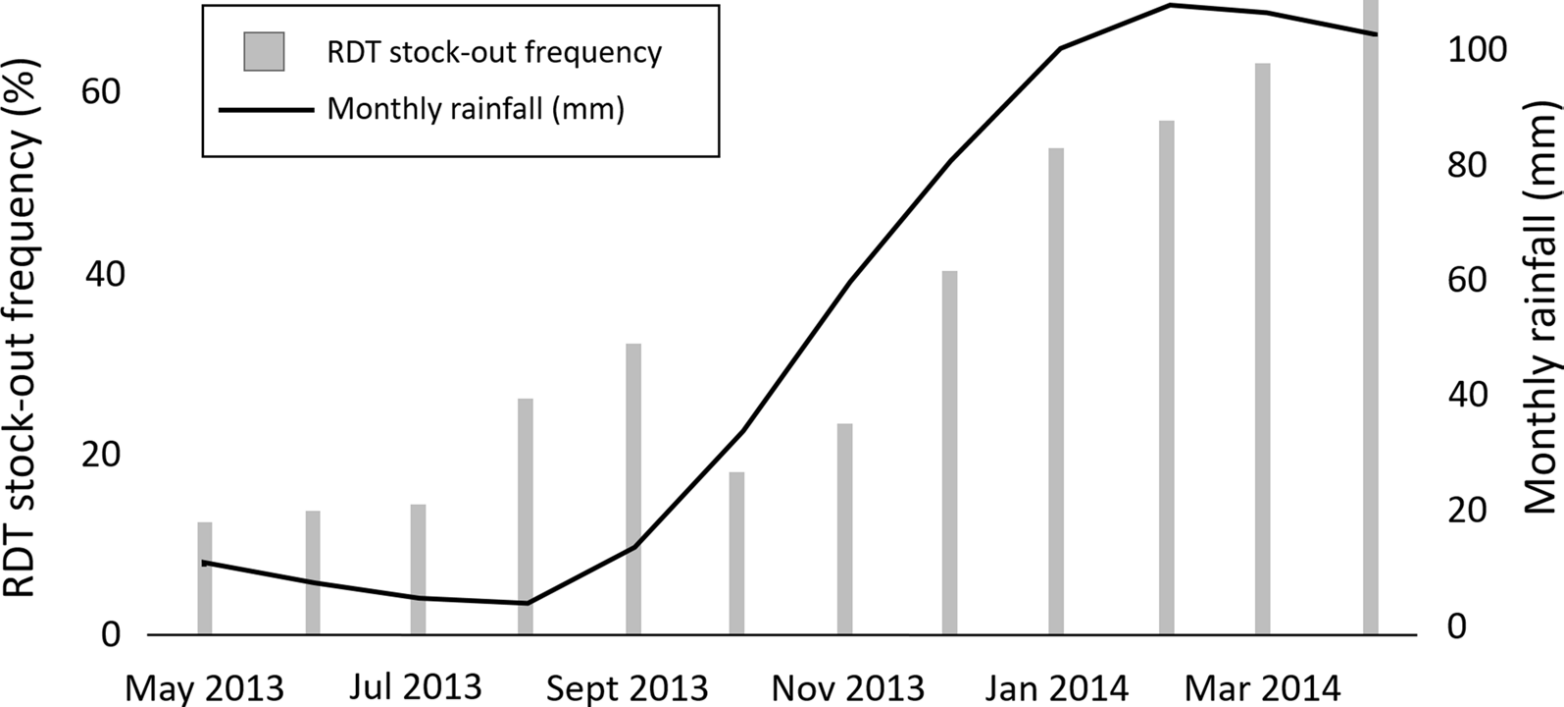
RDT stock-outs and monthly rainfall in Kondoa over a 12-month inventory period

All health facilities had at least one type of malaria test in stock, 35% (n = 14) of them had both RDTs and microscopes, whereas 58% (n = 23) had only RDTs and 8% (n = 3) only microscopes. On average, RDTs as diagnostic tools were available in 63% of the health facilities over a 12-month period, although their availability varied in time with an overall constant increase in the frequency of stock-outs between May 2013 and April 2014 (Fig. 1). RDT availability in government-owned health facilities was, on average, 64% over the 12-month period but only 44% during the rainy season (December to April) when malaria incidence increases. The majority of healthcare workers reported RDT stock-outs (n = 33; 83%) with delays in delivery being the most frequently stated reason.

RDT availability significantly varied between dispensaries (70%) and health centres (42%), i.e., RDTs were 3.2 times more likely to be available in dispensaries than in health centres (95% CI: 1.6–6.3; p < 0.001). The hospital and laboratory were excluded from categorization because there were too few observations (9 and 12, respectively). Comparisons between government versus faith-based organization ownership did not show any significant differences regarding RDT availability. Comparison with private facilities was not feasible owing to a lack of relevant information (e.g. seven observations from one health facility).

Information regarding BFM testing availability was incomplete because the physical inventory for BFM was only performed in 116 of the 309 data months. However, based on the available data, blood films and microscopes were available in 91% of health facilities and their availability was constant over time. Any further comparisons of microscope availability between categories of health facility level and ownership was not feasible owing to a lack of data.

### Survey with healthcare providers

#### Socio-demographic background of interviewees and setting

The majority of HCPs interviewed were females (63%) (Table 2). A total of 35% were nurses, followed by medical attendants (28%) and their mean work experience was 19.2 years (min. 7 months, max. 41 years). The great majority of HCPs were working in a dispensary (83%). HCPs interviewed declared that they performed laboratory diagnosis of malaria (93%), consulted patients (75%) and assisted in consultations (53%).

**Table 2:** Socio-demographic setting and activity profile of healthcare providers interviewed

	Observations (N = 40)		%
Age (years)			
Younger than 30	8		20.0
30–39	6		15.0
40–49	9		22.5
50 or older	17		42.5
Sex			
Female	25		62.5
Male	15		37.5
Profession			
Nurse	14		35.0
Medical attendant	11		27.5
Clinical officer	7		17.5
Laboratory assistant	5		12.5
Medical doctor	3		7.5
Work experience (years)			
Less than five	10		25.0
5–19	8		20.0
20–29	10		25.0
30 or more	12		30.0
Level of health facility			
Dispensary	33		82.5
Health Centre	4		10.0
District hospital	2		5.0
Private laboratory	1		2.5
Ownership of health facility			
Government	28		70.0
Faith-based	8		20.0
Private	4		10.0
Location of health facility			
Village	22		55.0
Ward headquarters	10		25.0
District town	8		20.0
Malaria-related tasks at health facility			
Laboratory diagnosis of malaria	37		92.5
Consultation of patients	30		75.0
Other	25		62.5
Assistance in consultation	21		52.5

#### Malaria perception in the area of service

Of the participating HCPs, 73% claimed to perceive malaria as a minor (or average) health concern, while only 28% answered that they considered malaria a major health concern. Among the latter, the two most cited reasons were the “high number of patients” (n = 10) and the “low knowledge of prevention” (n = 8). Among the 29 HCPs perceiving malaria as a minor concern, the most frequent reasons given were “high knowledge of prevention” (n = 26), “high knowledge of malaria” (n = 19), “low number of patients” (n = 18), “low risk of infection” (n = 16), “no common disease any longer” (n = 16) and “many negative results” (n = 11).

#### Training for malaria diagnostic

More than three quarters of respondents reported that they had received at least one type of training for the use of RDTs (n = 31; 78%). Twenty were trained by the district medical officer at the district level, 10 by laboratory technicians, 6 by co-workers, 3 at college, 1 by a clinical officer and 1 was trained in “another” way. In contrast, 20% (n = 8) did not receive any training and one person could not remember if they had received any such training. Out of the 30 HCPs who responded to a question concerning the number of times they received RDT training, the majority stated that they received training once (n = 25; 83%), two were trained twice and three received training on three or more occasions. The last time any of the 31 respondents received RDT training was on average 2.4 years before data collection commenced in 2014 (min. 1 years, max. 4 years). Finally, almost all respondents declared that they felt the need for further RDT training (n = 37; 93%). The most frequently cited reasons for needing further RDT training were “to improve the quality of the service” (n = 33), “to improve knowledge” (n = 27), “to improve the accuracy of the test result” (n = 24) and “to increase skills” (n = 19). Only 30% (n = 12) of the respondents received microscopy training for malaria diagnosis. At the time of data collection, the last time respondents were trained to use BFM for malaria diagnosis was on average 11.7 years earlier (min. 1 year, max. 39 years).

#### Knowledge, attitudes and perceptions towards malaria diagnosis

Inquiring on the preference of malaria testing method, almost half of HCPs (n = 19; 48%) claimed to prefer using microscopy for testing for malaria, while 43% (n = 17) preferred using RDT. Among the former, the three most frequently given reasons for their preference were “trust in blood slide” (n = 19; 100%), “old/well known test” (n = 12; 63%), the microscopic test being “more accurate” (n = 11; 58%) and “no trust in RDT” (n = 8; 42%). The most frequent reasons given for a stated preference for RDT were that they were “simple to use” (n = 16; 94%), they provide a “quick test and result” (n = 15; 88%), that they “trust in RDT” (n = 13; 77%) and that they are “more accurate” compared to microscopic diagnosis (n = 6; 35%). Specifically inquiring on trust, the majority of all HCPs (n = 35, 88%) declared that they trusted RDT results because (i) they trusted the accuracy of the test (94%); (ii) the accuracy was proven by the government (76%); (iii) it is easy to use (67%); and (iv) using RDTs was an “instruction by the government” 40% (n = 13). Five respondents (13%) stated that they did not trust the accuracy of the test result because of an issue with false negative results.

When considering only the HCPs that answered the question on test preference (36 out of 40), test preference seemed to vary significantly with the profession of the respondent (p = 0.046). Nurses, clinical officers and medical doctors tended to prefer RDT to microscopy testing (64%, 67% and 71%), whilst the majority of laboratory assistants and medical attendants preferred microscopy testing over RDT (60% and 90%, respectively). The stated preference also varied significantly by type of malaria tests available at the health facility (p = 0.014). Among those facilities where both tests were available, 77% of health workers stated a preference for RDT, whereas among those facilities where only RDT or only microscopy were available, 30% stated a preference towards RDT. If only one of the two diagnostic tests were available in the health facility, health workers were 87% less likely to prefer RDT compared with health workers of health facilities where both tests were available (OR: 0.13 [95% CI 0.019–0.761], p = 0.009). Among those who declared that they had never received RDT training (n = 8), 71% favoured RDT and 29% favoured microscopy. In contrast, among those who received RDT training (n = 28) the preference was less clear: 57% claimed to prefer microscopy and 43% claimed to prefer RDT.

Inquiring on the practice of confirming diagnostic test results: in the case of a positive RDT result, 48% of respondents reported that they would advise confirmation with microscopy, whereas 53% would not advise it (Table 3). The top reason for advising confirmation with blood films were “trust in blood slide” (95%), while the main reasons given for not advising confirmation with microscopy were “trust in result” (95%) and “trust in RDT” (86%). In the case of a negative RDT result, the majority of the respondents (68%) would advise confirmation with microscopy. The top reason for advising such confirmation was “trust in microscopy” (92%), while the main reasons for not advising so, were “trust in result” (100%), and “trust in RDT” (92%). HCPs who preferred RDT were six times more likely against advising confirmation of a negative RDT result with microscopy when compared with HCPs that preferred microscopy testing. Furthermore, all those who did not trust the accuracy of the RDT result (n = 5) advised confirmation of a negative RDT with microscopy. In the explicit case of a negative RDT result for a patient presenting with malaria symptoms, only 25% (n = 10) would confirm the negative result with a BFM test. Most (70%) would advise or perform a clinical diagnosis, 60% would not prescribe anti-malarial medication to the patient, 50% would give another medicine, 28% would use additional diagnostic tools, and 18% would prescribe anti-malarials anyway.

**Table 3:** Reasons stated for and against confirmation of a primary diagnosis

	Positive RDT, confirm with BFM?		Negative RDT, confirm with BFM?		Positive BFM, confirm with RDT?		Negative BFM, confirm with RDT?	
	Obs	%	Obs	%	Obs	%	Obs	%
No	21	52.5	13	32.5	26	65.0	27	67.5
Yes	19	47.5	27	67.5	14	35.0	13	32.5
Total	**40**	**100.0**	**40**	**100.0**	**40**	**100.0**	**40**	**100.0**
Yes: reasons								
Trust in BFM	18	94.7	24	92.3				
Trust in RDT					10	71.4	8	61.5
Other	15	78.9	16	61.5	5	35.7	6	46.2
Trust in both tests							6	46.2
No trust in result	7	36.8	11	42.3	4	28.6	4	30.8
No trust in RDT	4	21.1	5	19.2				
No trust in BFM					4	28.6		
BFM difficult to use					7	50.0	3	23.1
Total respondents	**19**	**100.0**	**26**	**100.0**	**13**	**100.0**	**13**	**100.0**
Total responses	**44**		**56**		**27**		**27**	
No: reasons								
Trust in result	21	100.0	13	100.0	24	92.3	26	96.3
Trust in RDT	18	85.7	12	92.3				
Trust in BFM					19	73.1	25	92.6
No trust in RDT					8	30.8	12	44.4
Trust in both tests	4	19.0	6	46.2	4	15.4	3	11.1
BFM difficult to use	2	9.5	4	30.8				
If symptomatic you give medicine	2	9.5	2	15.4			2	7.4
Other	2	9.5	1	7.7	8	30.8	6	22.2
Total respondents	**21**	**100.0**	**13**	**100.0**	**26**	**100.0**	**27**	**100.0**
Total responses	**49**		**38**		**63**		**74**	

*BFM* blood film microscopy, *RDT* malaria rapid diagnostic test

When HCPs were asked if they would advise confirmation of a positive microscopy result with RDT, the majority (65%) answered no (Table 3). The top reasons given for advising against confirmation with RDT were “trust in result” (92%) and “trust in blood slide” (73%), while the main reason given for advising for confirmation with RDT was “trust in RDT” (71%). When asked if they would advise confirmation of a negative blood film result with an RDT, the majority of the respondents (68%) answered no. The results were significantly associated with having received training for the use of RDT (n = 39; p = 0.01). Confirmation of a negative blood film result with RDT would not be advised by 77% of those having received RDT training versus 25% of those not having received RDT training (OR: 0.09 [95% CI: 0.009–0.747], p = 0.01).

Inquiring about how HCPs perceived healthcare-seekers’ preferences of malaria diagnostics, more than half of the staff believed that healthcare-seekers did not have a preference in any of the malaria diagnostic tests (n = 25; 63%). Of the remaining 33% (n = 15) believing that healthcare-seekers do have a preference, 80% felt that patients prefer microscopy testing, whereas only 20% felt that patients preferred RDT. The reasons given for justifying the answers were that patients had “trust in blood slide” (n = 12; 100%), patients had “no trust in RDT” (n = 8; 67%), patients would think that the “RDT result is always negative” (n = 7; 58%) and that patients have “no knowledge on RDT” (n = 3; 25%).

### Survey with household members

#### Characteristics of the households and household members

In total, 309 household members were interviewed, mostly from households located in a village 82%. A dispensary was the closest health facility for the majority of households (84%) and in 78% of cases, the closest health facility was government owned. More than half of the households (52%) were composed of 1–3 individuals, and the average household monthly income was 128,240 Tanzanian Shilling. Most respondents considered themselves either as the head of the household (47%) or as the husband/wife of the household head (42%). The mean age of the respondents was 40 years and the majority were females (68%). Most respondents were married or living together (76%), and 90% of respondents attended school with the highest level of education attained by 81% of respondents being primary education or post-primary training. The most-used source of information was the radio with 66% of respondents listening to it almost every day or at least once a week. One third (31%) read newspapers/magazines and 22% watched television, at the same frequency. A total of 89% of respondents contributed to their household cash income. The most frequently stated means of contribution were agriculture and livestock (73%) and petty business (37%). Please consult Additional file 1: Table S1 for full details of the household characteristics.

#### Malaria perception among the respondents and their knowledge, attitudes and practices towards malaria diagnostics

Malaria was considered by 66% of respondents to be a major health concern where they lived, 33% considered it a minor health concern and 1% did not know. Malaria perception was significantly associated with having ever attended school: malaria was perceived as a major health concern by 70% of the respondents that attended school and by 42% of those who did not (OR: 3.15 [95% CI: 1.38–7.31], p = 0.002). Malaria perception was also significantly associated with the place of living: malaria was perceived as a major health concern in their area of living by 86% of those living in a district town, by 58% of those living in ward headquarters and by 65% of those living in a village (OR (district town vs. others): 3.42 [95% CI: 1.13–13.9], p = 0.019).

Among the interviewees, 63% knew that BFM was a method to test for malaria, while 28% were familiar with the RDT and only 10% were aware of both testing methods. In total, 86% of interviewees said they would be interested in knowing more about the RDT test. The majority of respondents (96%) learned about malaria tests mainly from health facilities, 3% learned about the tests from the media and 0.3% through conversations with people.

Most respondents (68%) were last tested for malaria between two and seven months from the date of the interview. Government-owned health facilities appeared to be the most common places for the respondents to be last tested (n = 210, 68%) and the most common health facilities visited were dispensaries (39%) (Table 4). In accordance, when asked to which facility respondents would choose to visit if they had malaria symptoms, government health facilities would be chosen by 79% and 51% of respondents would go to a dispensary.

**Table 4:** Choice of health facility

	Observation	%
Health facility level tested for malaria the last time		
Dispensary	120	38.8
District hospital	107	34.6
Health centre	46	14.9
Private laboratory	36	11.7
Total	309	100.0
Health facility type tested for malaria the last time		
Government	210	68.0
Private	69	22.3
Faith-based	30	9.7
Total	309	100.0
Preferred health facility level when malaria symptoms present		
Dispensary	156	51.3
District hospital	89	29.3
Health centre	43	14.1
Private laboratory	16	5.3
Total	304	100.0
Preferred health facility type when malaria symptoms present		
Government	240	78.9
Private	37	12.2
Faith-based	27	8.9
Total	304	100.0

The most frequently cited reason for choosing the last health facility that a respondent attended was the availability of diagnostic services (n = 218, 70.6%) followed by the perception of “good services” (n = 184, 60%), that the facility was close by (n = 108, 35%) and the service was affordable (n = 64, 21%). Yet, in 73% of cases, the respondents were last tested for malaria in a health facility that was not the one closest to their household. Of the respondents that had a dispensary as the closest health facility to their household, 44% were tested for malaria there, whereas 35% preferred to go to the district hospital, 11% to a health centre and 11% to a private laboratory. Before going to the health facility where they were tested for malaria the last time, 14% of interviewees had visited another health facility which was in most cases a dispensary (81%) and government-owned (78%).

Prior to seeking treatment in a health facility, 75% of respondents did not take any medication. Among the 25% that did, 52% took Paracetamol, 16% Artemether and Lumefantrine (ALu)/Coartem, 13% Sulfadoxine-pyrimethamine (SP). Only 2% of the respondents had purchased an RDT for themselves before going to any health facility.

The most frequently used malaria test during the respondents’ last visit to a health facility was BFM (66%), whereas 34% were tested with an RDT and only one respondent was tested with both tests. The test results (n = 306) were positive for 71% of respondents; 73% of those tested using the BFM tested positive for malaria and 67% of those tested using a RDT tested positive for malaria. More than three quarters of respondents said they were highly satisfied with the treatment received after testing for malaria (76%), 20% were fairly satisfied and only 4% were not satisfied.

#### Preference of malaria testing methods

More than half of the respondents (58%), if presented with malaria symptoms, stated that they would prefer to be tested by BFM, whereas 23% would rather be tested with an RDT and 18% did not have a particular preference. Only two respondents stated that they would like to be tested with a combination of both tests and two respondents did not know.

Among those having a preference for microscopy, the four most frequently given reasons were “high diagnostic standard” (84%), “trust in test/result” (61%), the interviewee “knows only this one test” (28%) and it is an “old test” (23%). The most frequently cited reasons for preferring RDT were that they provide a “quick result” (80%), that it is a “quick service” (67%) and RDTs are perceived as a “new/modern test” (30%).

#### Trust in blood film microscopy

There was a high level of trust in the accuracy of test results among those respondents that were last tested for malaria with BFM (96%). The most cited reasons for trusting the test results were that they had received a “correct treatment plan” (71%), that they had “trust in the test” (53%), that they received a “positive test result” (50%) and that it “confirmed assumptions” (33%). The most cited reasons for not trusting the test result were that they had received a “negative result” (75%), that “symptoms remain” (75%) and it “did not confirm an assumption” (63%). When asked whether they would get tested again with BFM, 86% of interviewees said that they would.

#### Trust in RDT

The level of trust in the test result was very high (87%) among those that were last tested for malaria with an RDT. The most frequently stated reasons for trusting the test results were that they had received a “correct treatment plan” (63%), a “positive test result” (43%), that they “trust in the test” (40%) and it “confirmed an assumption” (35%). The most frequently given reasons for not trusting the RDT result were that they had no trust in the results (79%), having received “negative results” (43%) and that the “symptoms remain” (43%).

As was the case for malaria diagnosis with BFM testing, a proportion of those that declared to have trust in the RDT result said they would not get tested with RDT again. Paradoxically, the most frequently mentioned reason for not wanting to get tested again with RDT was the delayed result (64%).

#### Behaviour following test results

Among the respondents who, at the beginning of the interview, said they were only aware of RDT as a malaria diagnostic tool, 82% would seek a second opinion if the test result was negative. In contrast, if the test result was positive only 9% of the respondents would seek a second opinion. Similarly, the vast majority (83%) of those who were only aware of BFM diagnostics would seek a second opinion in the case of a negative blood film result, whereas only 9% would do so in the case of a positive blood film result. Among those familiar with both malaria tests, 90% would seek a second opinion with a negative RDT and 20% would do so in the case of a positive RDT result. For BFM testing, 45% would seek a second opinion if faced with a negative blood film result and 10% of respondents would seek a second opinion in the case of a positive diagnostic result.

## Discussion

The perception of malaria as a major health concern differed remarkably between the interviewed HCPs and healthcare-seekers in the district of Kondoa. Whereas the majority of household interviewees perceived malaria as a major health concern, in accordance with the Malaria Indicator Survey from 2012 [48], less than one third of HCPs had the same opinion. The latter perceptions could be considered a reflection of the reduction in malaria incidence in Tanzania from 343.7 per 1000 population at risk in 2000 to 122.1 per 1000 population at risk in 2012 [49].

According to the health facilities’ logbooks, more than 20% of all malaria diagnoses relied solely on a clinical diagnosis between 2013 and 2014 thus increasing the likelihood in this group of being over-diagnosed with malaria and incorrectly treated with anti-malarials when suffering with non-malaria febrile illnesses that express similar symptoms to malaria [15]. Furthermore, there were a higher number of patients tested by BFM (n = 7795, 45%) with a much higher percentage of patients testing positive for malaria (71%) compared to those tested with RDT (n = 5709, 33%) with a much lower rate of malaria positivity (14%). Considering the challenges of microscopy as a diagnostic tool for malaria in rural Tanzania, the substantial number of patients that tested positive with BFM would have exacerbated the number of incorrect malaria diagnoses and treatments prescribed together with the potentially false positive clinical diagnoses [17, 18]. On the other hand, the relatively low proportion of malaria positive rates among the RDT tested individuals may contribute in fuelling concerns over the extent of false negatives that are still being observed on Mainland Tanzania and Zanzibar [50, 51]. While not perceived a major immediate concern, it was stressed that clinicians should be aware of the risk. Moreover, Bakari et al. recently suggested a need for surveillance of the status of pfhrp2 (and pfhrp3) gene deletions in Tanzania. Encoding histidine-rich protein 2 (HRP2)—the target protein for the RDT adopted by the Tanzanian NMCP—such gene deletion would increasingly lead to false negatives. Such deletions have been, indeed, reported in neighbouring Kenya and Rwanda and there is evidence of sporadic occurrence of pfhrp2/3 gene deletions in some areas of Tanzania [52]. Comparing the diagnostic tools used on the last visit to a health facility for a malaria test, according to the responses of household members that were interviewed, BFM was used in 66% of cases with 72% of those testing positive, a similar proportion of positive tests to the data collected from health facility logbooks between 2013 and 2014. The proportion of patients tested with BFM was very high considering that the majority of respondents (68%) were tested for malaria in a government-owned health facility, where the Ministry of Health guidelines indicated that patients should be tested for malaria with RDTs. Household interviewees reported receiving an RDT in only 34% of cases on the last visit to a health facility for a malaria test, and 67% of those reported having a positive test result. Albeit the overall percentage of RDTs reported here were similar to the retrospective health facility data collected, the percentage of positive RDT results, according to interviewees on their last health facility visit, was much higher than the 14% positivity result retrieved from the health facilities records. The only explanation for this is a potential bias at the moment of data collection and that a number of negative RDT test results may have been given a clinical malaria diagnosis and the respondents then stated a positive RDT result.

The average availability of RDTs over the 12-month period in all facilities was sub-optimal (63%). Focusing on the government-owned health facilities only, they produced a slightly better average of 64% over the 12-month period but a concerning 44% average availability during the rainy season when malaria incidence increases, as does the demand for RDTs.

The decreasing availability of RDTs in health facilities over the 12-month period, due to delayed deliveries and stock-outs among others, is likely to have contributed to HCPs using clinical diagnosis and BFM as a means of testing for malaria to cope with demands [36].

Where more than half of the HCPs thought that patients had no malaria diagnostic preference, the majority of patients (81%) expressed a clear preference for BFM. Less than a third had any knowledge of RDT as a malaria diagnostic tool. Although the level of trust in BFM was extremely high (96%) among those that were last diagnosed with BFM and the level of trust in RDT was high among those last diagnosed with RDTs, 75% of BFM interviewees and 79% of RDT interviewees would not trust their respective results if they presented as negative. Equally, the results showed that for those who had knowledge of both tests, the level of trust was highly influenced by the test result. A negative result was more likely to be accepted if it was diagnosed with a microscope compared to a negative result from an RDT and in the latter case there would be a stronger wish for confirmation of a negative test result.

Less than half of healthcare providers preferred using BFM as a trusted testing method, 57% of those considered it more accurate than an RDT and 42% did not trust RDTs. Conversely, less than half of HCPs preferred the use of RDTs mainly due to their simplicity and speed, whereas 77% of those considered they were more accurate than BFM. Although the majority of HCPs trusted the RDT test result, 70% would provide a clinical diagnosis in the case of a negative RDT with symptoms typical for malaria. This behaviour could be a reflection of conformity to much older and more confusing malaria diagnosis and treatment guidelines from 2006 that suggested using clinical judgement when a test result was negative [36, 53, 54]. However, given the many challenges HCPs face adhering to treatment guidelines in Tanzania [11, 19, 55], a complexity of other more tangible reasons could be considered more compelling, such as a need to provide a diagnosis and treatment and patient pressure to provide one [15, 19]. In cases where HCPs could confirm RDT results with a blood film, the majority would do so, particularly if the RDT result was negative (68%). In contrast, where HCPs could confirm BFM test results with an RDT, approximately two-thirds would not, whether the blood slide presented a positive or negative result. Although the question was hypothetical (i.e. not observed and documented in this study), the HCPs responses led to the assumption that they have higher trust in blood film results compared to RDT results and generally have a higher level of trust if results are positive. Of course, this difference in opinion between HCPs’ confidence of one testing method over another, despite treatment guidelines advocating different diagnosis and treatment, could be confusing to patients and potentially have a profound influence over their choice and trust in a particular method of testing. Non-conformity to malaria treatment guidelines and compliance to negative RDT results by HCPs in Tanzania will probably continue to be a problem until the capacity of HCPs to diagnose other febrile illnesses peculiar to a locality, paired with increased options for laboratory tests to rule out other reasons, has improved [10, 11, 15]. Continued and more focused training and supportive supervision of HCPs, especially when new treatment guidelines are introduced, should include the diagnosis and treatment of non-malaria febrile illnesses that include area specific illnesses. This can help provide HCPs with options to treat a patient that test negative for malaria and prescribe more suitable drugs to prevent the over use of malaria medication to help combat resistance to malaria medication and over use of limited resources. Positive uniform messages, reinforced from frequently trained HCPs, in addition to public behaviour change approaches could provide patients with a sense of security in accepting and adhering to WHO guidelines on malaria approved by the Tanzanian Ministry of Health [33].

### Limitations

The presented study was conducted in Kondoa district, which differs in malaria prevalence, ethnic composition, geography and income sources compared to other districts in Tanzania. Due to these differences and a relatively small sample size among healthcare workers (n = 40), results cannot be attributed to the overall population of Tanzania.

Household members were included in the study if tested for malaria within the last 12 months, causing the potential for recall bias. To decrease the possibility of bias, interviewees were probed by showing them a blood film and RDT device in case of uncertainty at the beginning of the interview. To reduce recall bias for selected questions providing multiple reply options, interviewees were first asked to answer spontaneously and following, reply options were read out. To reduce interviewer bias, on the other hand, data collectors received intensive training prior to the study commencement, which included a pilot study.

Questions relating to the confirmation of test results were hypothetical and not validated by observations and replies and therefore need to be interpreted with caution. Paper-based questionnaires have the potential to decrease data quality due to data entry mistakes. To ensure data entry of the highest quality, data was entered twice by a team of two.

Finally, the study was concluded in 2014, therefore, the article presents data that may not accurately reflect the current situation. Nevertheless, subsequent observations, the authors’ continued experience in Tanzania and the recent scientific literature indicate that many of the challenges found in 2014 remain until today, such as prevalent self-medication with anti-malarials in certain patient groups and a persistent lack of adherence to malaria testing guidelines by healthcare workers [11, 56]. Correspondingly, many of the conclusions drawn from this work, still hold true today and much of the insight generated still offer guidance, particularly when trends reverse or bottlenecks arise. Moreover, the current study fills an important gap in the scientific literature owing to the scarcity of relevant studies in this area across Tanzania and may serve as a comprehensive orientation to future interventions in the clinical management of malaria—most notably in the area of diagnostics and adherence to standard treatment guidelines. To the authors’ knowledge, this is still the only study that comprehensively covers the knowledge, attitudes and practices towards malaria diagnostics among both healthcare providers and healthcare-seekers in Tanzania.

## Conclusions

Frequent malaria and non-malaria febrile illness diagnosis and treatment training for HCPs is essential for healthcare providers to offer uniform, quality services that patients can trust. This requires regular RDT training for rural HCPs in addition to reinforcing good microscopy practice where they are used in quality assured conditions. HCPs adherence to the latest guidelines for treating patients presenting with malaria symptoms needs to be encouraged whether or not a negative test result ensues. As private health facilities and laboratories prove popular for malaria testing, all governmental and private health facilities require a uniform approach that could be directed by guidelines and/or by law. Malaria diagnosis and treatment guidelines, therefore, may need to be simplified and made available in Swahili for the lower cadres of HCPs to comprehend and advocate to the public. The onus on the public to adhere to correct diagnoses and treatments can also be reiterated and reinforced by tailored communication and behaviour change interventions with various forms of media and outreach that can target groups that are more in need, namely the poor and illiterate. Provided RDT distribution is strengthened to avoid stock outs, especially in the malaria peak season, these measures could have the potential of reducing the high number of clinically diagnosed malaria cases and inadequate quality microscopy diagnoses, thereby reducing anti-malarial prescriptions where they are not necessary and supporting the fight against anti-malarial resistance. These findings emphasize the need for a sensitive approach when introducing new diagnostic tools. It shows the need for the continuous provision of delivering knowledge and training for HCPs to establish the needed trust in test results and sharing of information for healthcare-seekers at the community level.

## Supplementary Information

**Additional file 1** **Table S1.** Characteristics of the household.

## Data Availability

The datasets used and/or analysed during the current study are available from the corresponding author on reasonable request.

## Abbreviations

RDT: Malaria rapid diagnostic test
HCP: Healthcare provider
WHO: The World Health Organization
Tanzania: The United Republic of Tanzania
ACT: Artemisinin-based combination therapy
HPSS: The Health Promotion and System Strengthening Project
OR: Odds ratios
CI: Confidence interval
Swiss TPH: Swiss Tropical and Public Health Institute
SDC: Swiss Agency for Development and Cooperation
BFM: Blood film microscopy
NIMR: National Institute of Medical Research
EKNZ: Ethikkommission Nordwest- und Zentralschweiz
ICF: Information and Consent Form
