# Supplementary material for: Knowledge, attitudes and practices towards malaria diagnostics among healthcare providers and healthcare-seekers in Kondoa district, Tanzania: a multi-methodological situation analysis
Source: Malar J. 2022 Jul 21;21:224. doi: 10.1186/s12936-022-04244-0 (PMC9306200; doi:10.1186/s12936-022-04244-0)
Supplement: Supplementary file 1 — Additional file 1 Table S1. Characteristics of the household. [file 12936_2022_4244_MOESM1_ESM.docx]

**Table S1** Characteristics of the household

|  | **Observation** | **%** |
| --- | --- | --- |
| Place of residence |  |  |
| Village | 253 | 81.9% |
| District town/town council | 30 | 9.7% |
| Ward headquarter | 26 | 8.4% |
| Total | 309 | 100.0% |
| Number of permanent household members |  |  |
| 1–3 | 161 | 52.1% |
| 4–6 | 89 | 28.8% |
| 7 or more | 59 | 19.1% |
| Total | 309 | 100.0% |
| Household monthly income (TSh) |  |  |
| 100,000-199,000 | 82 | 26.5% |
| Less than 50,000 | 77 | 24.9% |
| 50,000–99,000 | 66 | 21.4% |
| 200,000–299,000 | 58 | 18.8% |
| 300,000 or more | 26 | 8.4% |
| Total | 309 | 100.0% |
| Which health facility is the closest to your home? (level) |  |  |
| Dispensary | 256 | 83.7% |
| Health center | 38 | 12.4% |
| Private laboratory | 8 | 2.6% |
| District hospital | 4 | 1.3% |
| Total | 306 | 100.0% |
| Which health facility is the closest to your home? (type) |  |  |
| Government | 240 | 78.4% |
| Private | 36 | 11.8% |
| Faith-based | 30 | 9.8% |
| Total | 306 | 100.0% |
| Position of the respondent in the household |  |  |
| Head | 145 | 46.9% |
| Wife or husband | 130 | 42.1% |
| Son or daughter | 15 | 4.9% |
| Parent | 13 | 4.2% |
| Brother or sister | 4 | 1.3% |
| Other relative | 2 | 0.6% |
| Total | 309 | 100.0% |
| Age of the respondent |  |  |
| Younger than 30 | 86 | 27.8% |
| 30–39 | 85 | 27.5% |
| 40–49 | 59 | 19.1% |
| 60 or older | 43 | 13.9% |
| 50–59 | 36 | 11.7% |
| Total | 309 | 100.0% |
| Sex of the respondent |  |  |
| Female | 210 | 68.0% |
| Male | 99 | 32.0% |
| Total | 309 | 100.0% |
| Marital status of the respondent |  |  |
| Married/living together | 235 | 76.1% |
| Never married/never lived together | 28 | 9.1% |
| Divorced/separate | 23 | 7.4% |
| Widow/widower | 23 | 7.4% |
| Total | 309 | 100.0% |
| Have you ever attended school |  |  |
| Yes | 277 | 89.6% |
| No | 32 | 10.4% |
| Total | 309 | 100.0% |
| What is the highest level of school you attended |  |  |
| Primary or post-primary training | 233 | 80.9% |
| Secondary or higher education | 42 | 14.6% |
| Pre-primary or no education | 13 | 4.5% |
| Total | 288 | 100.0% |
| Do you read a newspaper or magazine |  |  |
| Not at all | 126 | 40.8% |
| Less than once a week | 86 | 27.8% |
| At least once a week | 82 | 26.5% |
| Almost every day | 15 | 4.9% |
| Total | 309 | 100.0% |
| Do you listen to the radio |  |  |
| Almost every day | 158 | 51.1% |
| Not at all | 68 | 22.0% |
| At least once a week | 45 | 14.6% |
| Less than once a week | 38 | 12.3% |
| Total | 309 | 100.0% |
| Do you watch television |  |  |
| Not at all | 216 | 69.9% |
| Almost everyday | 41 | 13.3% |
| At least once a week | 26 | 8.4% |
| Less than once a week | 26 | 8.4% |
| Total | 309 | 100.0% |
| Do you contribute to your household cash income? |  |  |
| Yes | 276 | 89.3% |
| No | 33 | 10.7% |
| Total | 309 | 100.0% |
| How do you contribute to the household income? |  |  |
| Agriculture and livestock | 198 | 72.5% |
| Petty business | 100 | 36.6% |
| Employment | 19 | 7.0% |
| Other | 9 | 3.3% |
| Total | 273 | 100.0% |
